# Prognostic Factors and Long-Term Survival in Kaposi’s Sarcoma Patients: Results from a 28-Year Retrospective Cohort

**DOI:** 10.3390/medicina61040724

**Published:** 2025-04-14

**Authors:** Emre Hafızoğlu, Murat Bardakçı, Yakup Ergun, Irfan Karahan, Derya Demirtaş Esmer, Doğan Bayram, Fahriye Tugba Kos, Efnan Algın, Oznur Bal, Dogan Uncu

**Affiliations:** 1 Department of Medical Oncology, Afyonkarahisar State Hospital, 03100 Afyonkarahisar, Turkey; 2 Department of Medical Oncology, Gazi Yasargil Training and Research Hospital, 21100 Diyarbakır, Turkey; dr.muratbardakci@hotmail.com; 3 Department of Medical Oncology, Bower Hospital, 21100 Diyarbakır, Turkey; dr.yakupergun@gmail.com; 4 Department of Medical Oncology, Ankara Bilkent City Hospital, 06800 Ankara, Turkey; irfan_karahan@yahoo.com (I.K.); drderyademirtas@gmail.com (D.D.E.); tugbasan@yahoo.com (F.T.K.); efnanalgin@gmail.com (E.A.); dr_ozn@yahoo.com (O.B.); doganuncu@yahoo.com (D.U.); 5 Department of Medical Oncology, Gülhane Training and Research Hospital, 06010 Ankara, Turkey; drdoganb@gmail.com

**Keywords:** Kaposi’s sarcoma, mortality, survival

## Abstract

*Background and Objectives:* Kaposi’s sarcoma (KS) is a rare malignancy with limited prospective data. The aim of this study was to evaluate the clinical features and prognostic factors of KS in a cohort of patients treated at a single center. *Materials and Methods*: Records of 83 patients with KS were retrospectively analyzed. Patient demographics, clinical features, and treatments were analyzed. Univariate and multivariate analyses were performed to evaluate factors affecting overall survival (OS). *Results*: The median age of the cohort was 65 years, and 22.9% were female. The classical type of KS was the most common (84.3%), with the most common site of localization being the feet (30.2%). The 5-year and 10-year OS rates were 82.7% and 70.8%, respectively. Univariate analysis identified age, performance score (ECOG PS), lymph node involvement, and disease stage as significant prognostic factors. However, in multivariate analysis, only the ECOG PS remained a significant predictor of OS. *Conclusions*: KS is a condition that requires long-term follow-up, and performance status is particularly critical for patient survival. In addition to our findings, comprehensive prospective studies are still needed to better understand the factors influencing patient survival in KS.

## 1. Introduction

Kaposi’s sarcoma (KS) is a rare lymphoangioproliferative malignancy etiologically linked to human herpesvirus-8 (HHV-8) and characterized by its diverse clinical presentations and variable prognosis [1]. Four distinct variants are recognized: classic KS (CKS), endemic (African) KS, epidemic KS [associated with HIV (human immunodeficiency virus)/AIDS (acquired immunodeficiency syndrome)], and immunosuppression-related or iatrogenic KS (iKS). These subtypes differ in epidemiology, clinical behavior, and treatment response, reflecting the complex interplay of viral, immunological, and environmental factors in KS pathogenesis [2].

CKS, prevalent in Mediterranean and Eastern European populations, typically affects older adults, peaking in the sixth to seventh decades, with a historical male predominance that appears to be diminishing over time [3,4]. Endemic KS, more aggressive in nature, is primarily observed in sub-Saharan Africa [5], while iKS arises in the context of iatrogenic immunosuppression, such as post-transplantation or during chronic immunosuppressive therapy. Notably, switching from calcineurin inhibitors to mTOR inhibitors has been associated with the regression of iKS, highlighting the role of immune modulation in its course [2,6,7]. Epidemic KS, the most common malignancy among HIV-infected individuals, often presents with disseminated disease, and remains a significant global health challenge despite advances in antiretroviral therapy [8].

The treatment of KS depends on the type of KS, the extent and course of the disease, and patient symptoms. While maintaining quality of life, systemic treatment often aims to provide disease control and symptom relief (e.g., reduction in the number and size of lesions, treatment of bleeding, pain, and lymphedema). However, the treatment of KS still lacks an established gold standard. Moreover, prospective trials are rare, and experience relies mostly on retrospective CKS data [2].

Clinically, KS manifests as purplish, reddish-blue, or dark brown cutaneous lesions—ranging from macules to nodules—that may ulcerate, bleed, or become hyperkeratotic [2]. Dermoscopy has become increasingly valuable in assessing Kaposi’s sarcoma, especially for its ability to reveal characteristic vascular patterns. Among these, the so-called ‘rainbow pattern’—a multicolored phenomenon typically seen in nodular or papular lesions—may assist clinicians in distinguishing KS from other skin conditions with overlapping features [9]. The extent of involvement varies by subtype: CKS tends to follow an indolent course with predominantly cutaneous lesions, whereas epidemic and iKS variants frequently involve lymph nodes, mucosa, and visceral organs (e.g., lungs, gastrointestinal tract, and liver), posing greater therapeutic challenges [2]. Treatment strategies are tailored to disease extent and patient status, ranging from localized interventions (e.g., surgical excision, radiotherapy, and cryotherapy) for limited disease to systemic therapies (e.g., pegylated liposomal doxorubicin and paclitaxel) for advanced cases. Despite these options, no universal gold standard exists, and treatment decisions often rely on symptom palliation and quality-of-life preservation rather than curative intent [2].

The rarity of KS has hindered large-scale prospective studies, leaving retrospective analyses as the primary source of clinicopathological and prognostic insights. Existing data suggest that factors such as age, immune status, disease stage, and performance status influence outcomes, yet robust predictors of survival remain poorly defined. This knowledge gap underscores the need for comprehensive studies to elucidate the natural history and prognostic determinants of KS across its variants. In this context, we conducted a 28-year retrospective cohort study at a single tertiary center to evaluate the clinical features, treatment approaches, and prognostic factors affecting overall survival in KS patients. By analyzing a diverse cohort spanning classic, iatrogenic, and HIV-related KS, this study aimed to enhance understanding of this malignancy and inform future management strategies.

## 2. Materials and Methods

### 2.1. Patients and Research Design

We carried out this retrospective cohort study with data from patients diagnosed with KS between January 1995 and 2023 at our center. Histologic diagnosis required the presence of proliferative miniature vessels and tumor-like fascicles composed of spindle cells and a vascular network. Positivity for HHV-8 was confirmed, but not strictly necessary for diagnosis. Upon presentation, patients underwent physical examination and routine hematologic and chemistry analysis. We included patients aged 18 years or older with no second primary cancer and with histologically proven KS lesions. Nevertheless, patients with incomplete medical charts inconvenient to our study protocol were excluded. We extracted data from patients’ medical files. Accordingly, we evaluated data from 83 of 90 patients that satisfied our inclusion criteria.

Data were extracted from electronic and paper-based medical records, encompassing the following demographic and clinical variables: age at diagnosis, sex, Eastern Cooperative Oncology Group performance score (ECOG PS), comorbidities, HHV-8 status, serology for the hepatitis B virus (HBV), hepatitis C virus (HCV), and HIV, KS subtype (classic, iatrogenic, or HIV-related), lesion localization, disease stage, lymph node and visceral involvement, treatment modalities, and survival outcomes (date of death or last follow-up). Physical examination and routine hematologic/chemistry analyses were performed at presentation to assess baseline status.

Disease extent was classified as localized (confined to skin, oral/nasal cavity, or extremities, involving two or more body regions) or disseminated (extending to lymph nodes, gastrointestinal tract, bones, breasts, or lungs). iKS was defined as KS arising in an immunosuppressive context (e.g., post-transplantation or high-dose corticosteroid/immunosuppressive therapy), excluding patients with comorbidities unrelated to immunosuppression. Treatment approaches included surgical excision, cryotherapy, radiotherapy, and systemic chemotherapy. Due to inconsistencies in historical response assessment and a lack of standardized follow-up imaging, treatment response was not evaluated. Overall survival (OS) was calculated from the date of diagnosis to death from any cause or last follow-up; however, progression-free survival (PFS) data were unavailable due to incomplete progression documentation.

### 2.2. Statistical Analysis

Descriptive statistics were reported as frequencies and percentages for categorical variables and medians with ranges for continuous variables. Survival analysis was conducted using the Kaplan–Meier method, with OS estimates presented at 95% confidence intervals (CI). Differences in survival between groups were assessed with the log-rank test in univariate analyses. Variables with a *p*-value < 0.05 in univariate analysis were entered into a Cox proportional hazards regression model to identify independent predictors of OS. Due to the limited sample size, ECOG PS was dichotomized (0–1 vs. 2–3) and age was grouped as ≤65 years and >65 years based on the cohort’s median age. All statistical analyses were performed using SPSS version 25.0 (SPSS Inc., Chicago, IL, USA), with a *p*-value < 0.05 considered statistically significant.

### 2.3. Ethical Considerations

We carried out this study in accordance with ethical standards set forth by the institutional research committee and the 1964 Declaration of Helsinki. Additionally, we were granted ethical approval by the Clinical Research Ethics Committee No. 1 of Ankara City Hospital on 25 January 2023 with approval number E1-23-3224.

## 3. Results

### 3.1. Baseline Characteristics

A total of 83 patients were included in this study. The median age at diagnosis of these patients was 65 years (18–90 years). While 64 (77.1%) patients were male, 19 (22.9%) were female. In addition, the ECOG PS of 64 patients (74.7%) was discovered to be 0–1, and 43 patients (51.8%) had a comorbid disease at the time of diagnosis. Almost all patients (82, 98.8%) demonstrated pathological HHV-8 positivity. Additionally, we found CKS in 70 patients (84.3%), HIV-related KS in 6 patients (7.3%), and iKS in 7 patients (8.4%).

Among the iKS subgroup, immunosuppression stemmed from glomerulonephritis (*n* = 2), rheumatological diseases (*n* = 2), renal transplantation (*n* = 2), and lung transplantation (*n* = 1). Time to KS diagnosis post-immunosuppression ranged from 6 months to 9 years. Treatments included corticosteroids with cyclosporine, etoposide, methotrexate, hydroxychloroquine, tacrolimus, or mycophenolate mofetil. Cyclosporine A (CyA) therapy was switched to everolimus in two patients following the diagnosis of iKS; detailed regimen changes were incompletely documented in other patients. Two iKS patients with HCV seropositivity and visceral involvement (lung and kidney transplant recipients) died during follow-up, while the remaining five survived. In the HIV-related KS subgroup, five of six patients (83.3%) had disseminated disease, with lymph node involvement in two and visceral metastases (stomach, liver, and rectum) in three. One patient had localized disease. HBV seropositivity was noted in one patient with esophageal involvement, confirmed by biopsy.

At diagnosis, 67 patients (80.7%) had localized disease, and 16 (19.3%) had disseminated disease, including 9 with lymph node involvement alone, 6 with visceral and lymph node metastases, and 1 with esophageal involvement only. Patch/plaque histology was observed in 12 patients (14.5%), nodular/tumoral in 43 (51.8%), and was unknown for 28 (33.7%) due to incomplete records (Table 1).

### 3.2. Disease Management and Outcomes

Treatment modalities varied: 30 patients (36.1%) underwent surgical excision alone; 28 (33.7%) received additional localized therapies (e.g., cryotherapy or radiotherapy); and 25 (30.2%) received systemic chemotherapy, primarily ABV (*n* = 10, 12.0%), BV (*n* = 8, 9.6%), paclitaxel (*n* = 4, 4.8%), or pegylated liposomal doxorubicin (*n* = 1, 1.2%). Two patients (2.4%) received interferon. Recurrence or progression occurred in 36 patients (43.3%), including 2 of 6 HIV-related KS cases and 3 of 7 iKS cases, while 47 (56.7%) showed no progression during follow-up (Table 2).

**Figure 1:**
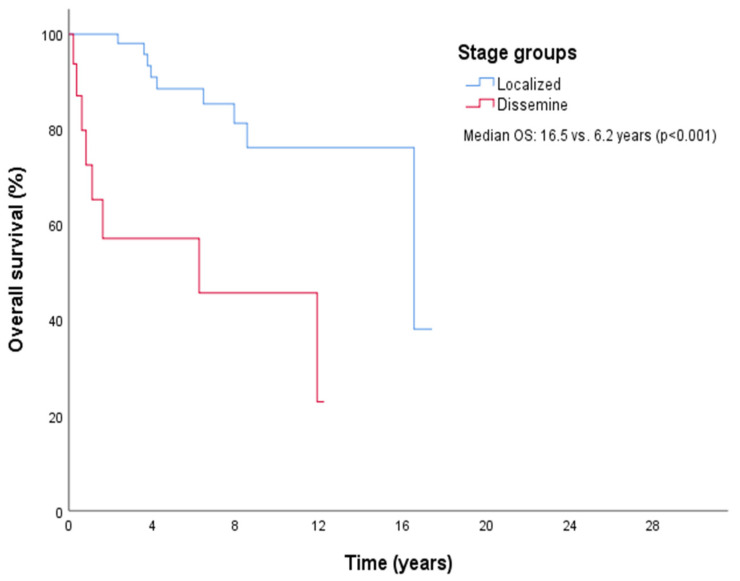
Kaplan–Meier curves for OS in groups by disease stage.

The median follow-up period was 4.6 years (range, 1.8–8.7). Median OS for the cohort was 16.5 years (95% CI: 9.9–23.0), with 5-year and 10-year OS rates of 82.7% and 70.8%, respectively. Seventeen patients (20.5%) died during follow-up. Subgroup analysis revealed a median OS of 16.5 years for CKS, 11.8 years for iKS, and not reached (NR) for HIV-related KS, although differences were not statistically significant (*p* = 0.51). Localized disease had a median OS of 16.5 years, compared to 6.2 years for disseminated disease (Figure 1).

**Figure 2:**
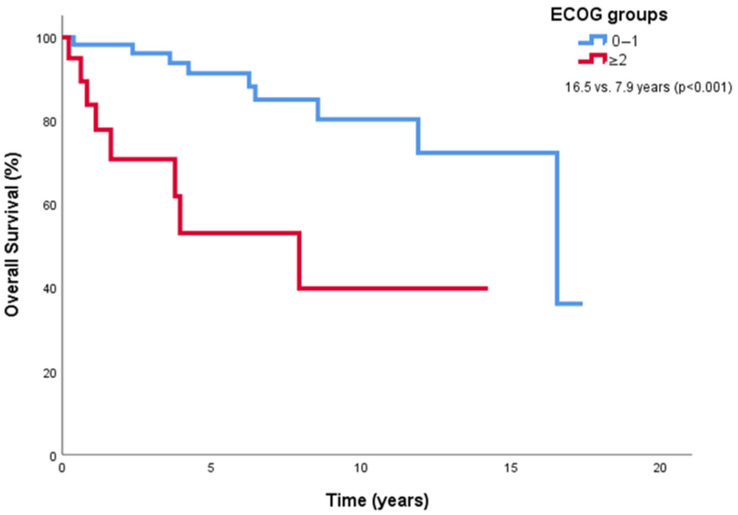
Kaplan–Meier curves for OS in groups by ECOG PS.

In univariate analyses, we evaluated sex, age, ECOG-PS, lymph node and visceral involvement at diagnosis, and disease stage. We grouped the age variable as ≤65 years and >65 years based on the median age (65 years). Due to the low number of patients, we grouped participants’ ECOG PS into two groups as “ECOG PS 0–1” and “ECOG PS 2–3.” Except for sex, all the variables above were predictors of poor survival. Next, these significant factors in univariate analyses were introduced in a Cox proportional-hazards regression model to identify independent prognostic factors. Consequently, only ECOG PS 0–1 was found to be an independent predictor of higher OS. Figure 2 presents Kaplan–Meier curves of overall survival by ECOG groups; Table 3 shows univariate and multivariate analyses of overall survival.

## 4. Discussion

KS is a rare malignancy, and its clinicopathological features and outcomes are often explored through retrospective studies due to the scarcity of prospective data. In this study, we investigated the demographic, clinical, and histopathological characteristics of KS, as well as treatment approaches and survival outcomes, in a single-center cohort followed over an extended period. Our findings offer insights into potential prognostic factors and survival patterns, contributing to the broader understanding of KS.

Our findings support the known association of KS with HHV-8 infection [1,2]. We detected positivity in 98.8% of the cases in our study, reinforcing its central role in KS development across its variants. Our demographic findings are consistent with prior epidemiologic data (predominant subtype, median age, male-to-female ratio, male predominance [7,10,11,12,13,14,15,16,17,18]. However, the presence of younger patients (24% under 50 years, with the youngest at 18 years) deviates from typical reports and may reflect our center’s status as a tertiary referral hospital, potentially attracting a broader demographic spectrum.

The lower extremities, particularly the feet (30.2%), were the most common sites of KS lesions in our cohort, corroborating previous findings [10,11,12]. However, we noted a higher frequency of systemic involvement (19.3% with disseminated disease) compared to some series, potentially attributable to the advanced disease states of patients referred to our center [10,11,12,13]. Among the iKS subgroup (8.4%), immunosuppressive regimens—most commonly corticosteroids—were implicated, with cases emerging as early as 6 months and as late as 9 years post-immunosuppression. This variability aligns with the recognized peak incidence within the first two years post-transplantation, although late-onset cases underscore the need for prolonged vigilance. In the HIV-related KS subgroup (7.3%), the younger median age (39.5 years) and higher rates of visceral involvement (50%) reflect patterns documented in the literature, although the absence of CD4 counts and antiretroviral therapy (ART) data limited deeper prognostic insights [10,14,15,19,20].

Treatment strategies in our cohort varied by KS subtype and disease extent. Localized therapies [19,20,21,22] (e.g., surgery, cryotherapy, and radiotherapy) were employed in 69.8% of cases, while systemic chemotherapy [1,23] (e.g., ABV, BV, and paclitaxel) was reserved for 28.8% of patients, primarily those with disseminated or symptomatic disease. The lack of standardized response criteria precluded treatment response evaluation, a limitation shared with many KS studies due to inconsistent definitions of clinical response (e.g., lesion size vs. number vs. symptom relief) [23,24,25]. This gap highlights the need for unified response assessment protocols in future research.

In this study, we found the median OS to be 16.5 years and realized that 17 patients died during follow-up. Additionally, 5- and 10-year OS rates were 82.7% and 70.8%, respectively. In their study, Orhan et al. reported the median OS to be 66.1 months and that half of the patients died [26]. Despite not reporting OS in their study, Yazıcı et al. documented the median follow-up to be 18.0 years and that 6.5% of patients died [14]. Yet, neither OS nor mortality rates were reported in other Türkiye-based studies [7,12,13,14,15,16,17,18]. On the other hand, the RARECARE study reported 5-year relative survival to be 83–86% [15]. In another study in Sweden, the 5- and 10-year OS rates were concluded to be 44% and 30%, respectively [27]. We think that the mentioned differences may have originated from focusing on different subgroups of KS and the varying numbers of participants included in these studies.

Univariate analyses identified age (>65 years), ECOG PS, lymph node involvement, visceral involvement, and disease stage as predictors of poor survival, consistent with prior research linking older age and immunosuppression to worse outcomes [19,28]. All variables that were statistically significant in the univariate analysis were re-analyzed, and no substantial collinearity was detected among them based on the variance inflation factor (VIF) and correlation matrix diagnostics. Therefore, all relevant variables were retained in the multivariate model. However, multivariate analysis isolated ECOG PS (0–1 vs. 2–3) as the sole independent predictor of higher OS (HR 7.45, 95% CI 2.23–24.85, *p* = 0.001). The loss of significance for other factors (e.g., stage, HR 0.94, *p* = 0.94) in multivariate analysis may reflect our limited sample size, which constrained statistical power, or unaccounted confounding variables. For example, the threefold survival difference between localized and disseminated stages in univariate analysis (16.5 vs. 6.2 years) suggests a strong prognostic signal diluted in multivariate modeling, warranting validation in larger cohorts.

This study’s limitations merit consideration. Its retrospective design precluded capture of critical data, such as CD4 counts in HIV-related KS or detailed immunosuppressive regimen adjustments in iKS, limiting subgroup analyses and the assessment of quality-of-life (QoL). Although subgroup analysis within the classic KS cohort may have provided additional insights, the limited number of cases restricted our ability to perform statistically meaningful comparisons. The small sample sizes of iKS (*n* = 7) and HIV-related KS (*n* = 6) further restricted statistical robustness, potentially underpowering comparisons across subtypes. Additionally, the inability to assess progression-free survival (PFS) or treatment response due to inconsistent historical records hampered evaluation of therapeutic efficacy. Cause-specific survival analyses were not adjusted for competing risks, which may have led to an overestimation of cancer-related mortality, particularly in elderly patients with multiple comorbidities. These gaps underscore the challenges of retrospective research in rare diseases and emphasize the need for prospective, standardized studies.

## 5. Conclusions

This 28-year retrospective analysis provides valuable insights into the clinical behavior and prognostic factors of KS. ECOG PS stands out as a significant predictor of survival, while other factors, such as disease stage, subtype, and treatment response, require further investigation. Our findings support the importance of a multidisciplinary approach and long-term follow-up in KS management, highlighting the need for future studies to focus on more homogeneous patient populations to better define prognostic and predictive factors.

## Figures and Tables

**Table 1:** Baseline characteristics.

Characteristics	*n* = 83 (%)
**Age (years) (median, min**–**max)**	65 (18–90)
**Age**	
** ≤65 years**	43 (51.8)
** >65 years**	40 (48.2)
**Sex**	
** Male**	64 (77.1)
** Female**	19 (22.9)
**ECOG PS**	
** 0**	36 (43.4)
** 1**	26 (31.3)
** 2**	15 (18.0)
** 3**	6 (7.3)
**Comorbidities**	
** No**	40 (48.2)
** Yes**	43 (51.8)
**Kaposi variants**	
** CKS**	70 (84.3)
** iKS**	7 (8.4)
** HIV-related KS**	6 (7.3)
**Histopathology stage**	
** Patch/plaque**	12 (14.5)
** Nodule/tumoral**	43 (51.8)
** Unknown**	28 (33.7)
**Immunocompromised condition**	
** Rheumatological disease**	2 (2.4)
** Glomerulonephritis**	2 (2.4)
** Renal transplantation**	2 (2.4)
** Lung transplantation**	1 (1.2)
**Viral status**	
** HBV**	1 (1.2)
** HCV**	2 (2.4)
** HIV**	6 (7.3)
**Primary location of KS and biopsy side**	
** Feet**	25 (30.2)
** Lower extremity except for feet**	14 (17.0)
** Upper extremity**	8 (9.6)
** Bilateral extremity**	12 (14.4)
** Generalized skin (extremity and trunk)**	10 (12.0)
** Head and neck**	7 (8.4)
** Oral cavity**	2 (2.4)
** Genital region**	2 (2.4)
** Jejunum**	1 (1.2)
** Esophagus**	1 (1.2)
** Breast**	1 (1.2)

ECOG PS: Eastern Cooperative Oncology Group performance score; HBV: hepatitis B virus; HCV: hepatitis C virus; HIV: human immunodeficiency virus; KS: Kaposi’s sarcoma; iKS: immunosuppression-related or iatrogenic KS.

**Table 2:** Staging, treatment, and survival.

**Stage at diagnosis**	
** Localized**	67 (80.7)
** Disseminated**	16 (19.3)
**Lymph node involvement at diagnosis**	
** No**	68 (81.9)
** Yes**	15 (18.1)
**Visceral involvement at diagnosis**	
** No**	76 (91.6)
** Bone**	1 (1.2)
** Breasts**	1 (1.2)
** Esophagus**	1 (1.2)
** Stomach**	1 (1.2)
** Liver**	1 (1.2)
** Jejunum**	1 (1.2)
** Rectum**	1 (1.2)
**Management strategies**	
** Surgery only**	30 (36.1)
** Excision and local treatment**	28 (33.7)
** Chemotherapy**	23 (28.8)
** Immunotherapy (IFN)**	2 (2.4)
**First-line chemotherapy drugs**	
** PLD**	1 (1.2)
** Paclitaxel**	4 (4.8)
** BV**	8 (9.6)
** ABV**	10 (12.0)
**Recurrence/progression**	
** No**	47 (56.7)
** Yes**	36 (43.3)
**Outcome**	
** Dead**	17 (20.5)
** Alive**	66 (79.5)
**Time to death, median (IQR), years**	16.5 (9.9–23.0)

IFN: interferon; PLD: pegylated liposomal doxorubicin; BV: bleomycin plus vincristine or vinblastine; ABV: adriamycin, bleomycin plus vincristine or vinblastine.

**Table 3:** Univariate and multivariate analyses of OS.

Variable	*n* (%)	Median OS (%95 CI)	Univariate Analysis HR (95% CI) *p* Value		Multivariate Analysis HR (95% CI) *p* Value	
**Sex**						
** Mal** **e**	64 (77.1)	16.5 (9.9–23.0)	1.17 (0.33–4.13)	0.79		
** Female**	19 (22.9)	NR				
**Age**						
** ≤65 years**	43 (51.8)	16.5 (9.9–23.1)	0.36 (0.12–1.03)	0.05		0.07
** >65 years**	40 (48.2)	NR				
**ECOG PS**						
** 0–1**	62 (74.7)	16.5 (9.9–20.1)	0.20 (0.07–0.54)	0.002	7.45 (2.23–24.85)	0.001
** 2–3**	21 (25.3)	7.9 (2.2–13.5)				
**Lymph node involvement**						
** No**	68 (81.9)	16.5 (5.3–27.7)	0.15 (0.05–0.41)	<0.001		0.93
** Yes**	15 (18.1)	6.2 (0.0–13.3)				
**Visceral involvement**						
** No**	76 (91.6)	16.5 (5.3–27.7)	0.31 (0.10–0.97)	0.04		0.63
** Yes**	7 (8.4)	11.8 (NR)				
**Stage**						
** Localized**	67 (80.7)	16.5 (5.3–27.7)	0.18 (0.07–0.49)	0.001		0.94
** Disseminated**	16 (19.3)	6.2 (0.1–14.9)				

ECOG PS: Eastern Cooperative Oncology Group performance score; HR: hazard ratio; CI: confidence interval.

## Data Availability

Due to ethical restriction rules of the instution and privacy, data sharing is not necessary.
